# Institutional evolution of a community-based programme for malaria control through larval source management in Dar es Salaam, United Republic of Tanzania

**DOI:** 10.1186/1475-2875-13-245

**Published:** 2014-06-25

**Authors:** Prosper P Chaki, Khadija Kannady, Deo Mtasiwa, Marcel Tanner, Hassan Mshinda, Ann H Kelly, Gerry F Killeen

**Affiliations:** 1 Ifakara Health Institute, Environmental Health and Ecological Sciences Thematic Group, Kiko Avenue, Mikocheni, PO Box 78373, Dar es Salaam, United Republic of Tanzania; 2 Vector Biology Department, Liverpool School of Tropical Medicine, Pembroke Place, Liverpool L3 5QA, UK; 3 Dar es Salaam City Council, Ministry of Regional Administration and Local Government, Dar es Salaam, United Republic of Tanzania; 4 Swiss Tropical and Public Health Institute, Basel, Switzerland; 5 Ministry of Science and Technology, Commission of Science and Technology (COSTECH), Dar es Salaam, United Republic of Tanzania; 6 Anthropologies of African Biosciences, Room 201, 15–17 Tavistock Place, London School of Hygiene and Tropical Medicine, London WC1H 9SH, UK; 7 Department of Sociology, Philosophy and Anthropology, University of Exeter, Exeter EX4 4PJ, UK

**Keywords:** Larval source management, Governance, Implementation, Mosquito, Malaria, *Anopheles*, *Plasmodium*, Community-based

## Abstract

**Background:**

Community-based service delivery is vital to the effectiveness, affordability and sustainability of vector control generally, and to labour-intensive larval source management (LSM) programmes in particular.

**Case description:**

The institutional evolution of a city-level, community-based LSM programme over 14 years in urban Dar es Salaam, Tanzania, illustrates how operational research projects can contribute to public health governance and to the establishment of sustainable service delivery programmes. Implementation, management and governance of this LSM programme is framed within a nested set of spatially-defined relationships between mosquitoes, residents, government and research institutions that build upward from neighbourhood to city and national scales.

**Discussion and evaluation:**

The clear hierarchical structure associated with vertical, centralized management of decentralized, community-based service delivery, as well as increasingly clear differentiation of partner roles and responsibilities across several spatial scales, contributed to the evolution and subsequent growth of the programme.

**Conclusions:**

The UMCP was based on the principle of an integrated operational research project that evolved over time as the City Council gradually took more responsibility for management**.** The central role of Dar es Salaam’s City Council in coordinating LSM implementation enabled that flexibility; the institutionalization of management and planning in local administrative structures enhanced community-mobilization and funding possibilities at national and international levels. Ultimately, the high degree of program ownership by the City Council and three municipalities, coupled with catalytic donor funding and technical support from expert overseas partners have enabled establishment of a sustainable, internally-funded programme implemented by the National Ministry of Health and Social Welfare and supported by national research and training institutes.

## Background

Indoor residual spraying (IRS) of houses with insecticides [1,2] and insecticide-treated nets (ITNs) [3] are the front line malaria vector control measures recommended across the globe and in Africa particularly [4]. However, outdoor feeding behaviours [5-7] and physiological resistance to insecticides [8-10] among residual vector populations define limits to what even these proven priority measures can achieve [11-14]. There has recently been a revival of interest in implementing and evaluating traditional larval source management (LSM) strategies to complement ITNs and IRS [15-21]. Some successful recent efficacy trials in rural Kenya [17,22] and Eritrea [23] have now been complemented by encouraging evidence of effectiveness in the context of the Dar es Salaam Urban Malaria Control Programme (UMCP) in Tanzania [24-26].

The pace of urbanization in Africa is the highest in the world; by 2030 at least half of this population is expected to live in towns and cities [27-29]. While these rapidly growing urban centres pose a number of public health problems [30], the high human population density and comparatively conducive infrastructural and governance conditions render LSM a more immediately feasible option for sustainable development than in rural contexts [31-33]. LSM has a long history of success in urban Africa, dating back almost 100 years [34,35]. Before World War II and the advent of modern adulticides for IRS and ITNs, combinations of environmental management, larviciding, mosquito-proofing houses, personal protection measures, and anti-malarial drugs were successfully applied to control malaria in an integrated fashion [36-38]. Urban malaria control in Tanzania during the 1960s relied heavily upon larviciding and community-implemented environmental management, such as drainage and habitat filling, resulting in malaria transmission that was considered to be of limited magnitude [39].

Community-based service delivery is considered vital to the effectiveness, affordability and sustainability of vector control generally [40-42], and to labour-intensive LSM programmes in particular [15,43-45]. The highly-localized task of detection and management of mosquito larval habitats encompasses public and private stakeholders at all spatial and governance scales. There is, therefore, a clear need to better understand the practices of governance that LSM necessitates, as well as the collaborative potential that exists between malaria-afflicted communities, research institutions and all levels of local and national government [44,45]. Participatory planning is essential to enhance local capacities and ensure community ownership, without which interventions usually fail because services remain under-utilized or misused [46,47]. However, the scope and extent of community participation usually remains poorly defined [48] and many have criticized utopian assumptions about the capacity of the ‘community’ to provide a panacea for a number of entrenched economic, social and health problems [49,50]. Others have questioned whether practices of ‘participation’ might, in fact, serve to diminish the democratic character of development, by limiting the ways in which citizenship is perceived [51,52].

This descriptive analysis examines the origins and evolution of a city-level LSM programme over 14 years in urban Dar es Salaam, Tanzania to better understand how such operational research projects can contribute to public health governance and establishment of sustainable service delivery programmes.

## Case description

### The Dar es Salaam Urban Malaria Control Programme (UMCP) in Tanzania

The overall goal of the contemporary Dar es Salaam UMCP is to reduce the incidence of malaria through the identification and treatment of the breeding grounds of *Anopheles* mosquitoes so that vector populations are substantially suppressed [45]. In order to realize this ambition, a portfolio of procedures for community-based monitoring and management of mosquito populations have been developed and evaluated [45,53,54]. The UMCP situates malaria control and associated operational research within the routine systems for municipal service provision by delegating the responsibility for larval control to community members known as Community Owned Resource Persons (CORPs) who are appointed through Street Health Committees across the city [45,53,55,56]. Between 2004 and 2009, the UMCP expanded effective LSM services across a substantial portion of the city, an area that includes fifteen wards and roughly 614,000 of the city’s three million residents [57]. At this scale, the UMCP is not only an operational research programme, but also a public health service delivery system of considerable size.

### Evolving institutional roles and responsibilities: tactical initiation to strategic development

**Figure 1:**
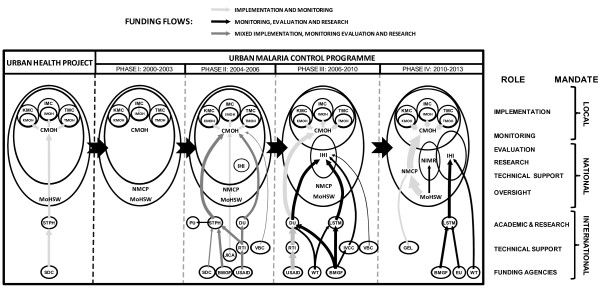
**The origin, developmental and subsequent reforms of responsibilities among partners and stakeholders of the UMCP, and the Urban Health Project in Dar es Salaam over the period described herein.** BMGF; Bill & Melinda Gates Foundation, CMOH; City Medical Office of Health, DU; Durham University, EU; European Union, GEL; Grupo Empresarial Labiofam; IHI; Ifakara Health Institute, IMC; Ilala Municipal Council, IMOH; Ilala Medical Office of Health, IVCC; Innovative Vector Control Consortium, JICA; Japan International Cooperation Agency, KMC; Kinondoni Municipal Council, KMOH; Kinondoni Medical Office of Health, LSTM; Liverpool School of Tropical Medicine, MoHSW; Ministry of Health and Social Welfare, NIMR; National Institute for Medical Research, NMCP; National Malaria Control Programme, PU; Princeton University, RTI; Research Triangle International, Swiss TPH; Swiss Tropical and Public Health Institute, TMC; Temeke Municipal Council, TMOH; Temeke Medical Office of Health, USAID; United States Agency for International Development, VBC; Valent Biosciences Corporation, WT; Wellcome Trust).

Several themes from the specific social and political history of Dar es Salaam define the origins and ontology of the UMCP. First, Tanzania’s colonial and postcolonial history suggests that ‘participation’ is a complex and highly resonant term signifying self-governance and/or the provision of labour. Second, an analysis of governance infrastructure in Dar es Salaam indicates that the particular spatial scale of civic engagement depends on distinct, correspondingly-scaled levels of political and administrative systems. Third, these formations and relationships are dynamic, evolving in response to social, administrative and institutional transformations. The health sector reforms of the 1990s in Tanzania were geared at empowering the district and municipal health services and their constituent communities in management and decision making [58,59]. Furthermore, the decentralization of the various operational processes at the municipal councils gave the various municipal bodies, including the Municipal Medical Offices of Health (MMOHs), autonomy in their functioning and responsibility to answer directly to their respective Municipal Health Management Boards, which are mandated to represent community interests. Rather than these report to the national Ministry of Health and Social Welfare (MoHSW), these health boards are immediately answerable to the Council management team – comprised of a total of 15 members including all heads of departments, Municipal Medical Officer, Municipal Pharmacist, to mention just a few, all under the oversight of the MoHSW. These reforms emphasised bottom-up management of health services and coincided with an international call to better understand and manage the effects of rapid urbanization upon health [60,61]. Consequently, the Urban Health Project (UHP), a bilateral programme between Tanzania and Switzerland, was initiated in Dar es Salaam in the early 1990s [62,63]. This project focused particularly on low-income urban populations and aimed at strengthening the health system as a whole. The implementation of UHP, which emphasized community participation, supported and extended local government reforms [63]. It was out of this framework that the UMCP evolved with a defined goal of staging community-based malaria control through LSM [45]. In keeping with the dynamic governance history of Dar es Salaam and Tanzania, the UMCP has gone through a series of developmental stages and reforms, notably characterised by increasingly well-defined allocation of operational responsibilities for the larvicide application and associated monitoring, evaluation and research activities to distinct stakeholder institutions (Figure 1).

### Partnering a local government initiative with national and international support institutions

With almost a century of relevant historical experience [34,64], a reformed and decentralized health system [58,62,63], policy support at national level [65], Dar es Salaam offered a particularly attractive programmatic context for developing community-based LSM at a time when the potential of these approaches had just returned to the international scientific agenda [37,66-68]. Nevertheless, the foundations of this current programme are local, rather than international or even national: The Ilala municipal council initiated a pilot community-based IVM programme covering seven wards in 2002, [45], even before LSM was re-integrated into the national malaria control priorities [65]. The fact that this local initiative was conceived by the council’s own planning team and was supported by the local government health budget, in the absence of specific funding support from the national treasury, particularly caught the attention of national and international research partners who shared an interest in developing a sustainable, community-based approach to urban malaria control [18,37,66-68]. A joint stakeholders’ meeting in Dar es Salaam in 2003 resulted in formulation of a joint plan for the first phase (Figure 1) of a modern, sustainable, community-based UMCP in Dar es Salaam [45].

Surveillance activities began in March 2004, followed by larval control activities two years later in three intervention wards where larvicide was routinely applied across an area of about 18.3 km^2^ with a population of over 128,000 residents [53]. This early roll-out proved to effectively reduce malaria prevalence by over 70% [24] at a cost of < $1 per person protected per year, comparing very favourably with gold standard interventions, such as LLINs and IRS [69]. Between 2007 and 2009, these implementation systems were sequentially scaled up to cover 15 out the 73 wards of Dar es Salaam with over 614,000 residents. This pilot programme for larvicide application was complemented by targeted drainage interventions in some of the mosquito-infested, low-lying valleys at the heart of the city [70,71] that were identified by the previous programme supported by the Japan International Cooperation Agency (JICA) the 1980s [34]. With the help of national and international experts and funding, the Bill & Melinda Gates Foundation (BMGF), the United States Agency for International Development (USAID) and the Swiss Agency for Development and Cooperation (SDC), the UMCP was established (Figure 1) as a community-based larval source management programme focusing particularly upon routine application of microbial larvicides for malaria control in urban Dar es Salaam. The programme was integrated into the vertical management and coordination portfolio of the City Medical Office of Health [45,53]. All the UMCP intervention and monitoring activities, such as participatory mapping, larvicide application and drain cleaning, as well as entomological monitoring of larval and adult stage mosquitoes, were implemented by community members engaged as Community-Owned Resource Persons (CORPs).

### Differentiating roles and responsibilities: wisdom in hindsight

Although the operational procedure was well outlined in the UMCP’s guidelines, the overall distribution of roles and responsibilities among the various local stakeholders were not clearly planned at the outset. Furthermore, funding for essentially all necessary implementation, monitoring, evaluation and operational research activities were channelled through a single, shared administrative mechanisms, inevitably resulting in competition for budget priority, and therefore friction, between the partners responsible for these distinct functions. These roles and responsibilities therefore had to be progressively clarified, refined and distinguished in practice during this second phase of UMCP (Figure 1). Consequently, the programme had to undergo significant adaptations in terms of redefining the organization and management roles of its local stakeholder institutions (Figure 1). The most important reform was the increasing separation of responsibilities for the main players on the ground, with the city and municipal councils increasingly focused upon implementation of larvicide application and day to day larval-stage mosquito surveillance while the Ifakara Health Institute (IHI) was increasingly tasked with operational research, monitoring and evaluation that included surveys of adult mosquito densities and malaria prevalence among residents. Furthermore, these increasingly well-defined collaborative and administrative relationships enabled more defined and effective allocation of funds for both research and implementation purposes.

Throughout the second and third phases of the UMCP, all relevant activities in Dar es Salaam relied upon channelling of donor funds through overseas institutions where most of the technical support partners were originally based (Figure 1). Initially funds from BMGF and USAID were channelled through Research Triangle International (RTI) and then the Swiss Tropical and Public Health Institute (STPH), respectively, from where some of it was apportioned to additional technical support partners at Princeton University (PU) and Durham University (DU). Furthermore, a pilot-scale environmental management evaluation, focusing upon clearing of existing drainage infrastructure [70,71], was directly administered and implemented by JICA in collaboration with the National Malaria Control Programme (NMCP) and the City Council. At the start of the programme, this arrangement gave the overseas technical support partners a high level of administrative authority and they correspondingly played a significant managerial role on the ground in Tanzania where one of the co-authors (GFK) was seconded by STPH on a full time basis. During this phase, personnel and funds for implementation, monitoring, evaluation and operational research were distributed through a single shared administrative system, team and programme office based at the Dar es Salaam city council. Shifting from this model into one where responsibilities and personnel were divided among various stake holders posed a considerable challenge; the upshot was that complementary implementation and technical support capacities could be developed separately and synergistically at appropriate national institutions.The third phase of UMCP was characterized by much better delineation of roles, responsibilities, funding and administrative systems of the national partner institutions (Figure 1). Third phase witnessed an increase in the number of donor partners, with the majority of funding coming from BMGF and USAID, but now supplemented with research and training grants from the Wellcome Trust (WT), European Union (EU) and Valent Biosciences Corporation (VBC). Essentially all implementation funds were channelled through RTI, then DU to support the implementation, monitoring and management activities of the city and municipal councils. A second administrative channel distributed funds through DU and, later on, through the Liverpool School of Tropical Medicine (LSTM) to support the operational research, monitoring, evaluation and training activities of IHI in support of local government partners. At this stage the local government partners were mainly tasked with implementation and monitoring roles with money managed directly by the City Council whereas the national level stakeholders such as IHI and the NMCP of the MoHSW were responsible for providing overall oversight, technical support, monitoring and evaluation (Figure 1). As the role and capacity of IHI as a national technical support partner grew during this phase, the role of overseas partners made a gradual transition from managerial to advisory. By the end of this third phase, the role of these external partners was largely restricted to technical advice, academic training and career support for the program. This marked a critical point in the evolution and growth of the UMCP into more than just a set of associated research projects but rather a functional programme with a strong collaborative national institutional base.

### Transition to a sustainable institutional and funding base

UMCP has entered its fourth phase in 2010, during which it’s governance structure and funding base was further improved (Figure 1). The successes of the UMCP [24,26] captured the attention of the Tanzanian government, which committed to finance all implementation activities of the UMCP as a programme of its own. Under this new funding scheme, UMCP has brought on board an important additional national technical support partners in the form of the National Institute for Medical Research (NIMR), tasked with additional monitoring and evaluation functions such as insecticide resistance testing [72,73], whereas the role of the MoHSW has been greatly strengthened by channelling these funds through the NMCP which oversees all aspects of the programme. Complementary research, monitoring and evaluation activities are now separately funded through competitive international research grants from the European Union, BMGF and WT and implemented by IHI so that technical expertise in the region has been strengthened and institutionalized. It is also critical to note that the institutionalization within IHI of most of the research and training capacity supporting the UMCP has enabled postgraduate training and career development for more than a dozen Tanzanian and Kenyan scientists and practitioners, registered at a diversity of academic partners in the region (University of Dar es Salaam, Sokoine University, University of Nairobi) and overseas (Swiss TPH, DU, LSTM).

### Roles and responsibilities of community participants in larval source management

Effective larval monitoring and control requires comprehensive knowledge of the urban landscape at remarkably fine spatial scales [74-77]. Like all African cities, Dar es Salaam is undergoing rapid growth, the majority of which is unplanned [78]. *Anopheles* habitats are particularly diverse, dynamic and unpredictable because of the high level of human activity, notably agriculture and construction [18,44,79-81]. Also, mosquitoes in cities continually and rapidly adapt to the peculiar selective pressures of urban environments so that their host-seeking behaviours [82], as well as their larval habitat preferences and tolerances, may differ from their better-studied rural counterparts [31,33]. Participatory learning through regular surveillance by community members [83] is therefore required for LSM programmes to react and adapt to highly dynamic and often surprising patterns of mosquito proliferation [43-45].

Sociologically speaking, human urban populations are typically far more diverse, dynamic and unstable, with higher rates of turnover, migration and crime [84]. As security and privacy are sources of concern, gaining access to the myriad of individual plots that comprise an African urban landscape poses one of the greatest challenges to effective surveillance, and presumably control, of larval-stage mosquitoes [56,85,86]. Recruiting participants through street-level committees was, therefore, of critical importance because only their familiarity with geography and residents of their neighbourhoods could enable location of and access to mosquito-breeding sites, many of which are located within private homes and gardens [85].

In contrast to the program sponsored by JICA in the late 1980s, the current UMCP delegates routine activities for both control and surveillance of mosquitoes to CORPs. While the CORPs working for the UMCP have always been trained and paid by the City Council, the sources of funding have varied over the years; initially relying upon external donors but now directly supported by the national treasury. CORPS are overseen by ward supervisors and recruited predominantly through neighbourhood health committees, which proved to be more effective than recruiting through centralized management systems [85,86]. Some pilot-scale environmental management activities were undertaken by the UMCP in selected low-lying locations with pre-existing but neglected drainage infrastructure where this approach was readily feasible [70,71]. However, for pragmatic reasons, the UMCP primarily focused upon developing, applying and evaluating generally applicable systems for comprehensive, routine application of biological larvicides [53].

### Community-based mapping

In order to enable rigorous support and supervision of CORPS responsible for implementing and monitoring larvicide application, a participatory mapping protocol [55,57] was developed that begins with sketch maps drawn by individual CORPs for each of the Ten Cell Unit housing clusters for which he or she was responsible. The involvement of specialist, non-community-based personnel from the centralized institutions (Figure 1) in this mapping process, only begins when a geographic technician or scientist from one of the technical support partners accompanied the CORP to his or her area to verify, correct and formalize the boundaries of these intuitive sketch maps. This process of quality control and integration into formal, centralized management systems relied upon aerial photographs as a common, intuitive and practical frame of geographic reference at the interface between the residents, CORP and centrally-employed specialist geographic technician [55,57].

### Community-based larval surveillance and control

Knowing where to search or apply larvicides is only part of the problem. Ultimately, this carefully mapped array of plots simply provides a geographic and administrative framework within which the tasks of larval control and surveillance can be assigned, monitored and managed [24,53,55]. Specifically, in the original implementation system described in detail elsewhere [53], every plot was to be visited weekly by one member of each of two distinct teams-first a CORP responsible for rigorous application of larvicide and then, within one or two days, a CORP responsible for surveying potential mosquito larval habitats and whether they contain aquatic-stage mosquitoes [53,85,86].

### Community-based surveillance of adult mosquito populations

Right from the outset, adult mosquito surveillance proved a major challenge to the UMCP which took several years to address. While the relatively low vector densities found in African cities are much more difficult to monitor than their rural counterparts, they are perfectly capable of mediating stable, endemic, self-sustaining malaria transmission. The usual challenges of monitoring sparse urban vector populations were further compounded in Dar es Salaam by the poor responsiveness of local mosquito populations to conventional mosquito trapping tools [87]. While home-grown innovation provided a novel trap with sufficient sensitivity for application in Dar es Salaam and beyond [87-90], this new technology only proved to have epidemiological predictive power when applied through an intensive and extensive community-based system that enabled monthly sampling of mosquitoes at a spatial resolution of approximately 0.27 km^2^[54]. Like the larval surveillance and control activities, effective deployment of this mosquito-trapping scheme relies upon affordable, practical mapping systems that link community-based implementation activities in the field to advanced geographic information systems at centralized supporting institutions.

### Scaling responsibilities: the complementary roles of communities and institutes

Echoing Ronald Ross’s recommendations [91]*,* the UMCP guidelines for the CORPs situate larval control within local land use and ownership:

*For the purposes of our programme, a plot is defined as a specific physical area with an identifiable owner, occupant, or user…Knowledge of who owns, occupies or uses a certain plot is very important if you are to gain unlimited and regular access in future as this is the person who has the power to say yes or no!*[53].

**Figure 2:**
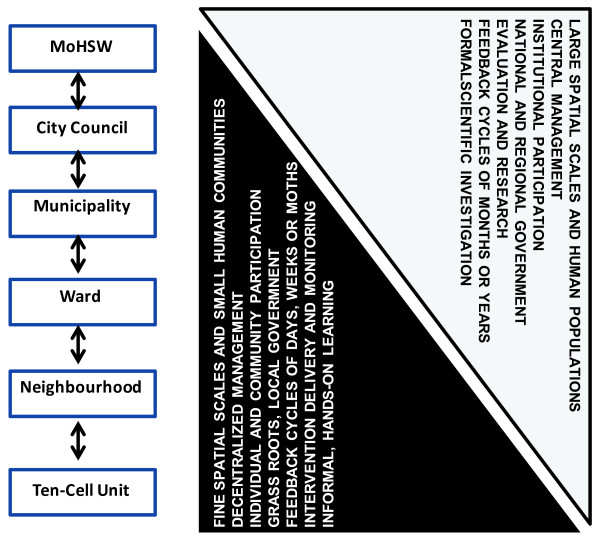
The scaling up and subsequent distribution of UMCP responsibilities among different stakeholders at the various administrative levels as well as spatial and temporal scales.

The fundamental geographic and administrative unit of larval control and surveillance by the UMCP is therefore the plot, embedded in social systems of regulation, and sometimes informal land markets, often beyond the purview of public authorities [92]. While the plot provides the *de facto* site of intervention, the city is the geographic unit of programmatic management and overall evaluation. This wider city scale is not only a matter of covering more ground but rather reflects the need for integration of these fine-scale units of decentralized implementation into large-scale systems of urban management and governance. The necessarily nested spatial structure of the UMCP introduces a diverse set of actors and correspondingly-scaled interactions between them (Figure 2).

This process of integrating such small individual plots into centralized local government support systems involved an iterative network of reportage: summaries, charts, spread sheets and reports connecting CORPs, Ward Supervisors, Municipal Inspectors, Municipal Coordinators and the City Council on a daily, weekly and monthly basis [53]. This system of annotated exchange enables the assessment of performance and evidence-based management at all the necessary spatial and temporal scales (Figure 2), detailing the roles of individuals and communities with those of their institutional partners [53]. Critically it allows those different administrative levels, from TCUs all the way up to the national MoHSW, to interact synergistically with each other while operating on corresponding fine or broad spatial and temporal scales. While effective management of mosquito populations begins on very fine scales with decentralized, community-based local management, the ultimate goal of achieving effectiveness at scale requires central management systems, funding, oversight and institutional support. The role of the institutional partners is to coalesce a myriad of otherwise independent, community-based management units into a single, stable coherent programme with consistent, evidence-based targets and monitoring systems, high-level governance stakeholders, and expert scientific support.

Of course, in practice, some degree of discord and friction between the various levels and partners are inevitable and perhaps even healthy and instructive. In Tanzania, popular participation has played a central role in colonial and postcolonial development schemes [93,94]. On the one hand, the familiarity of the idiom has given contemporary participatory approaches a peculiar traction. However, as the CORPs’ experience suggests, translation between disparate communities and the central institutions that support, service and mobilize them can result in a considerable amount of friction at their interface [85,86,95]. Understanding the diverse models and histories of participation is therefore particularly relevant in the context of larval control, as the space of intervention straddles the public, private, official and informal configurations of urban life.

### The community-local government interface

The grass roots workers, namely CORPs, *Mtaa* leaders and most ward supervisors, who comprise the vast majority of the programme’s personnel and implement most of the work on the ground, all work on a casual, very modestly-remunerated basis. In contrast to this, the municipal and city council officers that manage these teams are salaried, contracted government employees while the research partners enjoy even better employment conditions but assume no direct responsibility for the delivery of effective malaria control. Obviously, these disparities are most clearly felt by the CORPs responsible for the labour-intensive, day-to-day execution of the programme activities. Though they receive some compensation for their efforts, the value of these stipends is far less than the salary received by personnel formally employed by participating institutions. Moreover, payment through the established legal system for municipal mobilization of casual labour is hinged upon the completion of daily tasks, with no long-term security or provision for illness, bereavement or leave of any description. Though working for UMCP on what is legally considered a voluntary, casual basis, the time and sheer physical stamina it takes to locate and treat each and every potential breeding habitat across large areas means that the CORPs have limited opportunity to do any other work [86]. For most, participating in the UMCP was their primary, if not sole, source of income [86]. Compounding the difficulty of negotiating access to private residences, the demands placed on them by the UMCP were perceived by the CORPs as unrealistic and unfair [86].

Interestingly, despite their meagre remuneration, grass-roots personnel recruited as CORPs often performed routine activities on the ground better than salaried local government officers, and those recruited through local community leaders significantly outperformed those recruited through central management staff [86]. As the programme progressed, ward supervisor positions were increasingly filled by promoting local CORPs and awarding them a modest pay increase. Ultimately, the success of the programme depends on the capabilities and motivation of the CORPs to negotiate access to plots and to locate and treat larval habitats so the procedures for recruiting, managing and remunerating CORPs need re-evaluation. While larval surveillance CORPS engaged by local leaders were more successful at locating breeding habitats, their performance was generally poor and there may well be good reasons to hire smaller cadres of CORPs, who are fewer in number but better paid and incentivised [86].

### The researcher-implementer interface

The interface between local government implementers and their technical support partners also presents specific challenges that are critically important to overcome. First of all, the level of involvement of scientific staff in training, monitoring and management activities as technical advisors determines the fundamental nature of the evidence derived from impact evaluations [96] and these issues must be carefully managed throughout the development of such programmes. Where the level of such direct technical support is high, estimates of impact tend to reflect probabilistic evidence of efficacy under conditions which are less representative of sustainable scale up than would otherwise be the case [96]. Where a pilot evaluation is intended to form the nucleus of sustained public health programmes, and produce evidence of effectiveness under representative conditions of routine implementation, it is important to minimize such direct technical support and more clearly delineate the distinct and complementary roles of implementation and scientific partners [96].

The contemporary UMCP described here was initially established with research-based funding and a single programme office at which local government officials and scientists seconded from overseas worked together under one roof with poorly differentiated or defined roles (Figure 1). As the programme matured, a team of early-career Tanzanian scientists was established at a national research institute (IHI) operating from a separate office and the role of the expert partners from overseas shifted to providing a mixture of technical and academic support to national implementers and scientists with far more clearly distinguished roles and responsibilities (Figures 1 and 2). Often, health sector decentralization has been associated with a dramatic reduction in the number of health experts, and entomologists in particular, thus weakening internal capacity for monitoring of control operations at the various levels of central or local government [97-102]. In the case of the current UMCP, the reverse has been the case because it was rooted in a well-structured decentralized health programme that the UHP established, coupled with strong collaborations that existed between the local government and research partners. This integration allowed stable career development and growth of distinct professional cadres at these complementary and very different institutional bases: This partnership has witnessed a dozen Tanzanian and Kenyan researchers undertake PhD and MSc degrees while five local government employees graduated with MSc degrees in parasitology and vector ecology.

Of course the differentiation of such roles and responsibilities inevitable creates distinctions and interfaces that, in themselves, present substantive challenges to maintaining effective collaboration. Specifically, it must be recognized that it is extremely difficult for implementation partners to be entirely objective when assessing their own performance, as can be seen when one compares independent surveys of larval surveillance coverage and sensitivity [86] with internal monitoring data of the UMCP management system [53]. It is unreasonable to expect anyone to completely defy the natural pressures that arise from self-assessment so this is where the real value of independent scientific partners lies: to objectively and openly shed light on disappointing or frustrating features of implementation [56,85,86] while also lending credibility to encouraging evidence of success [24,26] through unbiased data collection, analysis and interpretation. The experience has been that maintaining these relationships is as strategically and vitally important as it is challenging. A lucid understanding of how these complementary roles are aligned, and long-term commitment to sustain them, is essential to cultivate on both sides of this interface and among high level oversight partners.

## Discussion and evaluation

Despite these collaborative challenges – not to mention the operational setbacks attendant to translating theory and good will into *de facto* public health practice – malaria prevalence and mosquito densities have consistently declined across the UMCP pilot area as larviciding has been sequentially scaled up [24,26]. Opportunities clearly exist for substantial improvement of many of the surveillance and intervention systems that comprise the UMCP [85,86]. While effective systems for safe, cost-effective community-based monitoring of adult vector populations on fine temporal and spatial scales have been developed and evaluated in Dar es Salaam, and could be applied elsewhere in other African LSM programmes [54], the same cannot yet be said for malaria incidence burden. Despite these limitations, the results of the earliest formal evaluation [24,26] were encouraging enough for the Tanzanian government through the MoHSW to decide to take over funding and management of the programme. The national government’s enthusiasm was shared by the Dar es Salaam City Council, which has expressed its commitment to expand the UMCP, from the fifteen wards where it was initially conducted, to all the urban and peri-urban areas of the city, and then all >90 wards of the city, by 2015. The UMCP faces many major challenges going forward, not least of which include the need to improve systems for larval surveillance and control [85,86] and extend its activities to tackle vectors of Lymphatic Filariasis, Dengue and Chikungunya. However, the key achievement is that is no longer a set of operational research projects but rather a nationally owned public health programme in the strict sense, so it will now have the opportunity to evolve, adapt and improve over the long term.

## Conclusions

The integration of the traditionally vertically-managed vector control activities into a decentralized community-based implementation system has achieved encouraging early success [24,26] and led to increased resources for wide-scale implementation of larval control in urban Dar es Salaam. The clear hierarchical structure associated with vertical organization of this community based and management systems, as well as the clear distinction in the lines of responsibilities across the various scales within UMCP, contributed to the evolution and subsequent growth of UMCP (Figure 2). Although the UMCP started off rather chaotically with the roles of the various partners ambiguously assigned (Figure 1), subsequent systematic separation of these roles and responsibility, as well as the central coordination role of the city council, enabled institutionalization of strengthened management and planning, improved community-mobilization capability, and capacity to exploit national and international funding systems. Ultimately, the high degree of programme ownership by the city council and three municipalities, coupled with catalytic donor funding and technical support from expert overseas partners have enabled establishment of a sustainable, internally-funded programme implemented by the national MoHSW through its NMCP and supported by national research and training institutes. Table 1 summarizes the lessons learned.

**Table 1:** Lessons Learned and recommendations from the large scale larval control program

**Serial number**	**Description of the specific lessons and recommendations**
1	*Operational research projects funded and scientifically supported by developed country partners should build upon local initiatives with existing stakeholders and advocates*
2	*All mosquito species must be targeted to reduce nuisance biting and maintain community support. Need to meet community expectations based on their perceptions of impact to whom the relationship between malaria, mosquito species and habitats is usually poorly understood in local communities who are often more motivated by mosquito biting nuisance than malaria or any other pathogens they transmit*
3	*A key challenge for mosquito control programs focusing on larviciding in urban areas is to have full, regular access to all open spaces potential for accommodating aquatic habitats where mosquito proliferation takes place, including all fenced plots and other areas with restricted access for the public. This requires substantive and open collaboration between stakeholders and residents.*
4	*Community involvement in both the recruitment process of the individuals and implementation of the intervention is therefore essential to programme performance*
5	*Wide-scale community-based implementation can be effectively achieved through a decentralized vertical management structure, utilizing the hierarchical gradient of implementation strategies and partner roles across all the necessary spatial scales. Such centralized coordination is essential to enable institutionalization of strengthened management and planning, improved community mobilization capability, and capacity to exploit national and international funding systems.*
6	*Effective mechanisms for communication and feedback of monitoring data within days, weeks or months rather than years are essential for LSM of mosquitoes that can develop from egg to adult within a week.*
7	*LSM requires continuous and thorough monitoring because success and failure occurs on remarkably fine spatial (< 1km2) and temporal scales (≤1 week) that match to the retreatment cycles and geographic division of responsibility to individual staff.*
8	*While surveillance of larval mosquito populations to assess the effectiveness of larvicide application, and the performance of individual personnel are essential internal monitoring functions, external quality assurance of these activities, as well as monitoring and evaluation of impact on adult mosquitoes and malaria risk should be separately conducted by an institutionally independent partners reporting directly to the programme management to avoid conflicts of interest that inevitably arise from self assessment.*
9	*Proven systems for rigorous and timely monitoring of LSM remain to be fully developed and take many years to slowly evolve to address the high standards required to ensure rapid identification of implementation failures at sufficiently fine spatial and temporal scales. For example, the decentralized, community-based use of a mosquito trap, which had to be specifically designed and optimized to address the local needs of the UMCP*[87,101,102]*, took 7 years to develop and evaluate*[54]*.*
10	*LSM programmes should therefore start small on manageable pilot scales and then progressively build and institutionalize their capacity and experience. Training and developments costs should therefore be included in budgets that are strategically planned and consistently supported over the long term so that locally-adapted LSM programmes and their supporting institutions have sufficient time to learn, consolidate and stabilize.*
11	*Such pilot programmes should follow clear, prospectively designed institutionalization plans that unambiguously delineate who will do what, at what spatial scale, and how the multiple independent institutions that are required will interact. Ambiguities regarding institutional roles and responsibilities inevitably results in i) competition between the technical and oversight partners, ii) politicization of the technical partners at the expense of doing their day-to-day technical work, and iii) a disconnect with the partners in other sectors, especially the local government.*
12	*Channeling funding for all necessary implementation, monitoring, evaluation and operational research activities through a single, shared administrative mechanisms inevitably results in unhealthy competition for budget priority between the partners responsible for these distinct functions. Each partner institute should administer its own distinct, pre-agreed, ring-fenced budget in a manner that prevents conflicts of interest, such as compromising the independence of external monitoring, evaluation and operational research activities by making the responsible partners contractually dependent upon the implementation partners they are obliged to assess objectively.*
13	*Because effectiveness of LSM programmes relies upon monitoring and managing at very fine spatial and temporal scales, the ability to collate, synthesize and report simple but reliable monitoring data in the shortest time possible are essential. Furthermore, maintaining and managing a stable funding base, as well as an effective collaboration between the partner institutions responsible for the diverse and distinct functions of an LSM programme are paramount to long term success. In most lower-income countries, capacity to manage logistics, human resources, institutional partnerships and funding support are most limiting, far more so at this juncture than technical entomology skills.*

## Competing interests

None of the funders had any role in the evaluation design, data collection, analysis, interpretation, drafting of the manuscript or decision to publish. None of the authors has any competing interests.

## Authors’ contributions

PPC conceived and led study design, literature review and drafting of the manuscript. HM, MT KK JKM and. AK supported the design and implementation of this study. HM, MT and GFK contributed substantially to drafting of the manuscript. GFK supervised all aspects of the study design and drafting of the manuscript. All authors read and approved the final manuscript.

## References

[B1] Kouznetsov RL Malaria control by application of indoor spraying of residual insecticides in tropical Africa and its impact on community health Trop Doctor 1977 7 81 93 10.1177/004947557700700216 854978

[B2] Pluess B Tanser FC Lengeler C Sharp BL Indoor residual spraying for preventing malaria Cochrane Database Syst Rev 2010 4 CD006657 20393950 10.1002/14651858.CD006657.pub2 PMC6532743

[B3] Lengeler C Insecticide-treated bed nets and curtains for preventing malaria Cochrane Database Syst Rev 2004 2 CD000363 15106149 10.1002/14651858.CD000363.pub2

[B4] WHO World malaria report 2013 2013 Geneva: World Health Organization

[B5] Govella NJ Chaki PP Killeen GF Entomological surveillance of behavioural resilience and resistance in residual malaria vector populations Malar J 2013 12 124 23577656 10.1186/1475-2875-12-124 PMC3637503

[B6] Russell TL Beebe NW Cooper RD Lobo NF Burkot TR Successful malaria elimination strategies require interventions that target changing vector behaviours Malar J 2013 12 56 23388506 10.1186/1475-2875-12-56 PMC3570334

[B7] Durnez L Mao S Denis L Roelants P Sochantha T Coosemans M Outdoor malaria transmission in forested villages of Cambodia Malar J 2013 12 329 24044424 10.1186/1475-2875-12-329 PMC3848552

[B8] Ranson H N'Guessan R Lines J Moiroux N Nkuni Z Corbel V Pyrethroid resistance in African anopheline mosquitoes: what are the implications for malaria control? Trends Parasitol 2011 27 91 98 20843745 10.1016/j.pt.2010.08.004

[B9] Trape JF Tall A Diagne N Ndiath O Ly AB Faye J Dieye-Ba F Roucher C Bouganali C Badiane A Malaria morbidity and pyrethroid resistance after the introduction of insecticide-treated bednets and artemisinin-based combination therapies: a longitudinal study Lancet Infect Dis 2011 11 925 932 21856232 10.1016/S1473-3099(11)70194-3

[B10] Edi CVA Koudou BG Jones CM Weetman D Ranson H Multiple-insecticide resistance in *Anopheles gambiae* mosquitoes: Southern Cote d'Ivoire Emerg Infect Dis 2012 18 1508 22932478 10.3201/eid1809.120262 PMC3437712

[B11] Griffin JT Hollingsworth TD Okell LC Churcher TS White M Hinsley W Bousema T Drakeley CJ Ferguson NM Basáñez MG Reducing *Plasmodium falciparum* malaria transmission in Africa: a model-based evaluation of intervention strategies PLoS Med 2010 7 e1000324 20711482 10.1371/journal.pmed.1000324 PMC2919425

[B12] Eckhoff P Mathematical models of within-host and transmission dynamics to determine effects of malaria interventions in a variety of transmission settings Am J Trop Med Hyg 2013 88 817 827 23589530 10.4269/ajtmh.12-0007 PMC3752743

[B13] Killeen GF Seyoum A Sikaala C Zomboko AS Gimnig JE Govella NJ White MT Eliminating malaria vectors Parasit Vectors 2013 6 172 23758937 10.1186/1756-3305-6-172 PMC3685528

[B14] Killeen GF A second chance to tackle African malaria vector mosquitoes that avoid houses and don't take drugs Am J Trop Med Hyg 2013 88 809 816 23589532 10.4269/ajtmh.13-0065 PMC3752742

[B15] WHO Global Strategic Framework for Integrated Vector Management 2004 Geneva: World Health Organization

[B16] WHO Malaria Vector Control and Personal Protection: Technical Report 2006 Geneva: WHO 1 72 16623084

[B17] Fillinger U Ndenga B Githeko AK Lindsay SW Integrated malaria vector control with microbial larvicides and insecticide treated nets in western Kenyan highlands: a controlled trial Bull World Health Organ 2009 87 655 665 19784445 10.2471/BLT.08.055632 PMC2739910

[B18] Killeen GF Fillinger U Kiche I Gouagna LC Knols BGJ Eradication of *Anopheles gambiae* from Brazil: lessons for malaria control in Africa? Lancet Infect Dis 2002 2 618 627 12383612 10.1016/s1473-3099(02)00397-3

[B19] Killeen GF Knols BG Gu W Taking malaria transmission out of the bottle: implications of mosquito dispersal for vector-control interventions Lancet Infect Dis 2003 3 297 303 12726980 10.1016/s1473-3099(03)00611-x

[B20] Fillinger U Lindsay SW Larval source management for malaria control in Africa: myths and reality Malar J 2011 10 353 22166144 10.1186/1475-2875-10-353 PMC3273449

[B21] Tusting LS Thwing J Sinclair D Fillinger U Gimnig J Bonner KE Bottomley C Lindsay SW Mosquito larval source management for controlling malaria Chochrane Database Syst Rev 2013 8 CD008923 10.1002/14651858.CD008923.pub2 PMC4669681 23986463

[B22] Fillinger U Lindsay SW Suppression of exposure to malaria vectors by an order of magnitude using microbial larvicides in rural Kenya Trop Med Int Health 2006 11 1629 1642 17054742 10.1111/j.1365-3156.2006.01733.x

[B23] Shililu J Mbogo C Ghebremeskel T Githure J Novak R Mosquito larval habitats in a semiarid ecosystem in Eritrea: impact of larval habitat management on *Anopheles arabiensis* population Am J Trop Med Hyg 2007 76 103 110 17255237

[B24] Geissbuhler Y Kannady K Chaki PP Emidi B Govella NJ Mayagaya V Kiama M Mtasiwa D Mshinda H Lindsay SW Tanner M Fillinger U de Castro MC Killeen GF Microbial larvicide application by a large-scale, community-based program reduces malaria infection prevalence in urban Dar es Salaam, Tanzania PLoS One 2009 4 e5107 19333402 10.1371/journal.pone.0005107 PMC2661378

[B25] Dongus S Chaki P Govella N Majambere S Kannady K Russell T Killeen GF Smithson P Community-based larviciding of mosquitoes contributes to malaria reduction IHI spotlight 2011 Dar es Salaam: Ifakara Health Institute

[B26] Maheu-Giroux M Castro MC Impact of community-based larviciding on the prevalence of malaria infection in Dar es Salaam, Tanzania PLoS One 2013 8 e71638 23977099 10.1371/journal.pone.0071638 PMC3743749

[B27] Cohen B Urban growth in developing countries: a review of current trends and a caution regarding existing forecasts World Dev 2004 32 23 51

[B28] Montgomery MR The urban transformation of the developing world Science 2008 319 761 764 18258903 10.1126/science.1153012

[B29] UN: United Nations Population Division World Urbanization Prospects: The 2001 Revision—Data Tables and Highlights 2002 New York: Population Division, Department of Economic and Social Affairs, United Nations Secretariat

[B30] Kjellstrom T Friel S Mercado S Havemann K Sattherthwaite D Our Cities our Health our Future: Acting on Social Determinants for Health Equity in Urban Settings 2007 Kobe, Japan: WHO Centre for Health Development Report to the WHO Commission on Social Determinants of Health from the Knowledge Network on Urban Settings

[B31] Keiser J Utzinger J Castro MC Smith TA Tanner M Singer BH Urbanization in sub-Saharan Africa and implication for malaria control Am J Trop Med Hyg 2004 71 Suppl. 2 118 127 15331827

[B32] Hay SI Guerra CA Tatem AJ Atkinson PM Snow RW Urbanization, malaria transmission and disease burden in Africa Nat Rev Microbiol 2005 3 81 90 15608702 10.1038/nrmicro1069 PMC3130901

[B33] Robert V MacIntyre K Keating J Trape JF Duchemin JB Warren M Beier JC Malaria transmission in urban sub-Saharan Africa Am J Trop Med Hyg 2003 68 169 176 12641407

[B34] Castro MC Yamagata Y Mtasiwa D Tanner M Utzinger J Keiser J Singer BH Integrated urban malaria control: a case study in Dar es Salaam, Tanzania Am J Trop Med Hyg 2004 71 Supplement 2 103 117 15331826

[B35] Clyde DF Malaria in Tanzania 1967 London: Oxford University Press

[B36] Lindsay SW Emerson PM Charlwood JD Reducing malaria transmission by mosquito-proofing homes Trends Parasitol 2002 18 510 514 12473368 10.1016/s1471-4922(02)02382-6

[B37] Utzinger J Tanner M Kammen DM Killeen GF Singer BH Integrated programme is key to malaria control Nature 2002 419 431 12368831 10.1038/419431a

[B38] Keiser J Singer BH Utzinger J Reducing the burden of malaria in different eco-epidemiological settings with environmental management: a systematic review Lancet Infect Dis 2005 5 695 708 16253887 10.1016/S1473-3099(05)70268-1

[B39] Clyde DF Malaria control in Tanganyika under the German administration-Part 1 East Afr Med J 1961 38 27 42 24546262

[B40] Heintze C Garrido MV Kroeger A What do community-based dengue control programmes achieve? A systematic review of published evaluations Trans R Soc Trop Med Hyg 2007 101 317 325 17084427 10.1016/j.trstmh.2006.08.007

[B41] Winch P Kendall C Gubler D Effectiveness of community participation in vector-borne disease control Health Policy Plan 1992 7 342

[B42] WHO Integrated Vector Control. Seventh report of the WHO Expert Committee on Vector Biology and Control 1983 Geneva: World Health Organ Tech Rep Ser 1 72 6414188

[B43] Townson H Nathan R Zaim M Guillet P Manga L Bos R Kindhauser M Exploiting the potential of vector control for disease prevention Bull World Health Organ 2005 83 942 947 16462987 PMC2626501

[B44] Killeen GF Mukabana WR Kalongolela MS Kannady K Lindsay SW Tanner M Cadlas de Castro M Fillinger U Habitat targetting for controlling aquatic stages of malaria vectors in Africa Am J Trop Med Hyg 2006 74 517 518 16606973

[B45] Mukabana WR Kannady K Kiama GM Ijumba JN Mathenge EM Kiche I Nkwengulila G Mboera L Mtasiwa D Yamagata Y van Schayk I Knols BG Lindsay SW Cadlas de Castro M Mshinda H Tanner M Killeen GF FU Ecologists can enable communities to implement malaria vector control in Africa Malar J 2006 5 9 16457724 10.1186/1475-2875-5-9 PMC1409792

[B46] WHO World Health Report: Working Together for Health 2006 Geneva: World Health Organization

[B47] Hongoro C McPake B How to bridge the gap in human resources for health Lancet 2004 364 1451 1456 15488222 10.1016/S0140-6736(04)17229-2

[B48] Rifkin SB Muller F Bichmann W Primary health care: on measuring participation Soc Sci Med 1988 26 931 940 3388072 10.1016/0277-9536(88)90413-3

[B49] Bhattacharyya J Solidarity and agency: rethinking community development Hum Organ 1995 54 60 69

[B50] Leach M Scoones I Wynee B Science and Citizens: Globalization & the Challenge of Engagement 2005 London and New York: Zed Books

[B51] Cooke B Kothari U Participation: The New Tyranny? 2001 London: Zed Books

[B52] Mosse D Cooke B Kothari U ‘People’s Knowledge’, Participation and Patronage: Operations and Representations in Rural Development 2001 London: Zed Books

[B53] Fillinger U Kannady K William G Vanek MJ Dongus S Nyika D Geissbuehler Y Chaki PP Govella NJ Mathenge EM Singer BH Mshinda H Lindsay SW Tanner M Mtasiwa D de Castro MC Killeen GF A tool box for operational mosquito larval control: preliminary results and early lessons from the Urban Malaria Control Programme in Dar es Salaam, Tanzania Malar J 2008 7 20 18218148 10.1186/1475-2875-7-20 PMC2259364

[B54] Chaki PP Mlacha Y Msellemu D A M Malishee AD Mtema ZJ Kiware SS Zhou Y Lobo NF Russell TL Singer BH Mshinda H Lindsay SW Tanner M Mtasiwa D de Castro MC Killeen GF An affordable, quality-assured community-based system for high resolution entomological surveillance of vector mosquitoes that reflects human malaria infection risk patterns Malar J 2012 11 172 22624853 10.1186/1475-2875-11-172 PMC3475008

[B55] Dongus S Nyika D Kannady K Mtasiwa D Mshinda H Fillinger U Drescher AW Tanner M Castro MC Killeen GF Participatory mapping of target areas to enable operational larval source management to suppress malaria vector mosquitoes in Dar es Salaam, Tanzania Int J Health Geogr 2007 6 37 17784963 10.1186/1476-072X-6-37 PMC2025588

[B56] Vanek MJ Shoo B Mtasiwa D Kiama M Lindsay SW Fillinger U Kannady K Tanner M Killeen GF Community-based surveillance of malaria vector larval habitats: a baseline study in urban Dar es Salaam, Tanzania BMC Public Health 2006 6 154 16776829 10.1186/1471-2458-6-154 PMC1534019

[B57] Dongus S Mwakalinga V Kannady K Tanner M Killeen G Participatory mapping as a component of operational malaria vector control in Tanzania Geospat Anal Environ Health 2011 4 321 336

[B58] Mtasiwa D Seigfreid G Tanner M Pichette P The Dar es Salaam City/Region Minimum Package of Health and Related Management Activities: from Managing Diseases to Managing Health Systems 2003 Dar es Salaam: Dar es Salaam City Medical Office of Health 216

[B59] Anonymous Tanzania Joint Health Review 2003 Dar es Salaam: Ministry of Health & Social Welfare, United Republic of Tanzania 15

[B60] Knudsen AB Slooff R Vector-borne disease problems in rapid urbanization: new approaches to vector control Bull World Health Organ 1992 70 1 1568273 PMC2393336

[B61] WorldBank World Development Report 1993. Investing in Health: World Development Indicators 1993 16 London: Oxford University Press

[B62] Tanner M Urban Health in Developing Countries: Progress and Prospects 1995 London: Earthscan Publications

[B63] Harpham T Few R The Dar Es Salaam Urban Health Project, Tanzania: a multi-dimensional evaluation J Public Health Med 2002 24 112 119 12141579 10.1093/pubmed/24.2.112

[B64] Kilama WL Malaria in Tanzania: past and present Proceedings of the 11th Annual Joint Scientific Conference with a Seminar on Malaria Control Research 1994 Arusha, Tanzania: National Institute for Medical Research

[B65] MOH National Malaria MediumTerm Strategic Plan, 2002–2007 2002 Dar es Salaam: Ministry of Health United Republic of Tanzania & World Health Organization 55

[B66] Killeen GF Fillinger U Knols BGJ Advantages of larval control for African malaria vectors: low mobility and behavioural responsiveness of immature mosquito stages allow high effective coverage Malar J 2002 1 8 12153709 10.1186/1475-2875-1-8 PMC117646

[B67] Killeen GF Following in Soper's footsteps: northeast Brazil 63 years after eradication of *Anopheles gambiae* Lancet Infect Dis 2003 3 663 666 14522266 10.1016/s1473-3099(03)00776-x

[B68] Utzinger J Tozan Y Singer BH Efficacy and cost-effectiveness of environmental management for malaria control Trop Med Int Health 2001 6 677 687 11555434 10.1046/j.1365-3156.2001.00769.x

[B69] Worrall E Fillinger U Large-scale use of mosquito larval source management for malaria control in Africa: a cost analysis Malar J 2011 10 338 22067606 10.1186/1475-2875-10-338 PMC3233614

[B70] Castro MC Kanamori S Kannady K Mkude S Killeen GF Fillinger U The importance of drains for the larval development of lymphatic filariasis and malaria vectors in Dar es Salaam, United Republic of Tanzania PLoS Neglect Trop Dis 2010 4 e693 10.1371/journal.pntd.0000693 PMC2876116 20520797

[B71] Castro MC Tsuruta A Kanamori S Kannady K Mkude S Community-based environmental management for malaria control: evidence from a small-scale intervention in Dar es Salaam, Tanzania Malar J 2009 8 57 19356246 10.1186/1475-2875-8-57 PMC2683857

[B72] Kabula B Tungu P Matowo J Kitau J Mweya C Emidi B Masue D Sindato C Malima R Minja J Susceptibility status of malaria vectors to insecticides commonly used for malaria control in Tanzania Trop Med Int Health 2012 17 742 750 22519840 10.1111/j.1365-3156.2012.02986.x

[B73] Kabula B Tungu P Malima R Rowland M Minja J Wililo R Ramsan M McElroy PD Kafuko J Kulkarni M Distribution and spread of pyrethroid and DDT resistance among the *Anopheles gambiae* complex in Tanzania Med Vet Entomol 2013 doi: 10.1111/mve.12036 10.1111/mve.12036 PMC10884793 24192019

[B74] Orenstein AJ Contribution to the study of the value of quininization in the eradication of malaria J Am Med Assoc 1914 63 1931 1933

[B75] Watson M African Highway: The Battle for Health in Central Africa 1953 London: John Murray

[B76] Shousha AT Species-eradication. the eradication of *Anopheles gambiae* from Upper Egypt, 1942–1945 Bull World Health Organ 1948 1 309 353 20603927 PMC2553915

[B77] Soper FL Wilson DB Anopheles gambiae in Brazil: 1930 to 1940 1943 New York: The Rockefeller Foundation

[B78] Amer S Towards Spatial Justice in Urban Health Services Planning 2007 Enschede, The Netherlands: University of Utrecht

[B79] Dongus S Urban vegetable production in Dar es Salaam, Tanzania: GIS-supported analysis of spatial changes from 1992 to 1999 Appl Physiogeography Trop Subtrop Rep 2001 12 100 144

[B80] Kiunsi Pelling E, Winser B Building disaster-resilient communities: Dar es Salaam, Tanzania Disaster Risk Reduction: Cases from Urban Africa 2009 London: Earthscan

[B81] Dongus S Nyika D Kannady K Mtasiwa D Mshinda H Gosoniu L Drescher AW Fillinger U Tanner M Killeen GF Castro MC Urban agriculture and *Anopheles* habitats in Dar es Salaam, Tanzania Geospat Health 2009 3 189 210 19440962 10.4081/gh.2009.220

[B82] Govella NJ Okumu FO Killeen G Insecticide treated nets can reduce malaria transmission by mosquitoes which feed outdoors Am J Trop Med Hyg 2010 82 415 419 20207866 10.4269/ajtmh.2010.09-0579 PMC2829902

[B83] van den Berg H Knols BGJ The farmer field school: a method for enhancing the role of rural communities in malaria control Malar J 2006 5 3 16423295 10.1186/1475-2875-5-3 PMC1382236

[B84] Bang YH Shah NK Human Ecology related to urban mosquito-borne diseases in countries of the south-east Asia region J Commun Dis 1988 20 1 17 2906646

[B85] Chaki P Govella N Shoo B Abdullah H Tanner M Fillinger U Killeen GF Achieving high coverage of larval-stage mosquito surveillance: challenges for a community-based mosquito control programme in urban Dar es Salaam, Tanzania Malar J 2009 8 311 20042071 10.1186/1475-2875-8-311 PMC2806382

[B86] Chaki PP Dongus S Fillinger U Kelly A Killeen GF Community-owned resource persons for malaria vector control: enabling factors and challenges in an operational programme in Dar es Salaam, United Republic of Tanzania Hum Resour Health 2011 9 21 21955856 10.1186/1478-4491-9-21 PMC3204271

[B87] Govella N Chaki P Mpangile J Killeen G Monitoring mosquitoes in urban Dar es Salaam: Evaluation of resting boxes, window exit traps, CDC light traps, Ifakara tent traps and human landing catches Parasit Vectors 2011 4 40 21418622 10.1186/1756-3305-4-40 PMC3069960

[B88] Wong J Bayoh N Olang G Killeen GF Hamel MJ Vulule JM Gimnig JE Standardizing operational vector sampling techniques for measuring malaria transmission intensity: evaluation of six mosquito collection methods in western Kenya Malar J 2013 12 143 23631641 10.1186/1475-2875-12-143 PMC3648405

[B89] Sikaala CH Killeen GF Chanda J Chinula D Miller JM Russell TL Seyoum A Evaluation of alternative mosquito sampling methods for malaria vectors in lowland south east Zambia Parasit Vectors 2013 6 91 23570257 10.1186/1756-3305-6-91 PMC3639086

[B90] Sikaala C Chinula D Chanda J Hamainza B Mwenda M Mukali I Kamuliwo M Lobo NF Seyoum A Killeen GF A cost-effective, community-based, mosquito-trapping scheme that captures spatial and temporal heterogeneities of malaria transmission in rural Zambia Malar J 2014 13 225 24906704 10.1186/1475-2875-13-225 PMC4060139

[B91] Ross R Mosquito Brigades and How to Organize Them 1902 London: General Books LLC Publication

[B92] Kombe WJ Kreibich V Reconciling informal and formal land management: an agenda for improving tenure security and urban governance in poor countries Habitat Int 2000 24 231 240

[B93] Pratt C The Critical Phase in Tanzania, 1945–1968: Nyerere and the Emergence of a Socialist Strategy 1976 London: Cambridge University Press

[B94] Samoff J The bureaucracy and the bourgeoisie: decentralization and class structure in Tanzania Comp Stud Soc Hist 1979 21 30 62

[B95] Dongus S Pfeiffer C Metta E Mbuyita S Obrist B Building multi-layered resilience in a malaria control programme in Dar es Salaam, Tanzania Prog Dev Stud 2010 10 309 324

[B96] Habicht JP Victora CG Vaughan JP Evaluation designs for adequacy, plausibility and probability of public health programme performance and impact Int J Epidemiol 1999 28 10 18 10195658 10.1093/ije/28.1.10

[B97] Buchan J Health sector reform and human resources: lessons from the United Kingdom Health Policy Plan 2000 15 319 11012407 10.1093/heapol/15.3.319

[B98] Dovlo D Using mid-level cadres as substitutes for internationally mobile health professionals in Africa: A desk review Hum Resour Health 2004 2 7 15207010 10.1186/1478-4491-2-7 PMC455693

[B99] Kritski AL Ruffino-Netto A Health sector reform in Brazil: impact on tuberculosis control country case report Int J Tuberc Lung D 2000 4 622 626 10907764

[B100] Zimmerman RH Ecology of malaria vectors in the Americas and future direction Mem I Oswaldo Cruz 1992 87 371 383 10.1590/s0074-02761992000700064 1343717

[B101] Govella NJ Chaki PP Geissbuhler Y Kannady K Okumu F Charlwood JD Anderson RA Killeen GF A new tent trap for sampling exophagic and endophagic members of the *Anopheles gambiae* complex Malar J 2009 8 157 19602253 10.1186/1475-2875-8-157 PMC2720981

[B102] Govella NJ Moore JD Killeen GF An exposure-free tool for monitoring adult malaria mosquito populations Am J Trop Med Hyg 2010 83 596 20810826 10.4269/ajtmh.2010.09-0682 PMC2929057

